# Changing features of the Northern Hemisphere 500-hPa circumpolar vortex

**DOI:** 10.3389/fdata.2022.1009158

**Published:** 2023-01-09

**Authors:** Nazla Bushra, Robert V. Rohli, Chunyan Li, Paul W. Miller, Rubayet Bin Mostafiz

**Affiliations:** ^1^Department of Oceanography and Coastal Sciences, College of the Coast and Environment, Louisiana State University, Baton Rouge, LA, United States; ^2^Coastal Studies Institute, Louisiana State University, Baton Rouge, LA, United States

**Keywords:** tropospheric circumpolar vortex, 500-mb flow patterns, steering atmospheric circulation, fast Fourier transform, breakpoint analysis, climate change

## Abstract

The tropospheric circumpolar vortex (CPV), an important signature of processes steering the general atmospheric circulation, surrounds each pole and is linked to the surface weather conditions. The CPV can be characterized by its area and circularity ratio (*R*_*c*_), which both vary temporally. This research advances previous work identifying the daily 500-hPa Northern Hemispheric CPV (NHCPV) area, *R*_*c*_, and temporal trends in its centroid by examining linear trends and periodic cycles in NHCPV area and *R*_*c*_ (1979–2017). Results suggest that NHCPV area has increased linearly over time. However, a more representative signal of the planetary warming may be the temporally weakening gradient which has blurred NHCPV distinctiveness—perhaps a new indicator of Arctic amplification. *R*_*c*_ displays opposing trends in subperiods and an insignificant overall trend. Distinct annual and semiannual cycles exist for area and *R*_*c*_ over all subperiods. These features of NHCPV change over time may impact surface weather/climate.

## Introduction

The tropospheric circumpolar vortices (CPVs, Waugh et al., 2017) are the two hemispheric-scale, extratropical circulations that circumnavigate each polar region—one in the Northern Hemisphere (NHCPV) and the other in the Southern. The CPVs are positioned near the albeit discontinuous surface polar front at a given time, situated near the steepest geopotential height gradient, the sharpest gradient of air temperature, and the fastest flow of the quasi-westerly wind belt, including the polar front jet stream. Spatial features of the NHCPV area and circularity were examined by Bushra and Rohli (2019, 2021a), and the spatial and temporal features of NHCPV centroid position were described by Bushra and Rohli (2021b).

The amplitude and length of poleward (i.e., ridges in the Northern Hemisphere) and equatorward (i.e., troughs in the Northern Hemisphere) meanders in the Rossby waves in the CPV's westerly flow support surface storminess (Di Capua and Coumou, 2016) through upper-level divergence (ULD) and cyclonic vorticity advection (CVA), and suppress surface storminess by upper-level convergence (ULC) and anticyclonic vorticity advection (AVA). Likewise, ULC and AVA (ULD and CVA), driven by the Rossby waves, support (suppress) surface anticyclones. While the changes in the CPV's centroid location and dimensions (i.e., area and wave amplitudes in the form of ridges/troughs) are likely to affect the location and degree to which surface storms or anticyclones beneath the CPV are supported, surface features may also influence the CPV properties (Angell and Korshover, 1985; Kirby et al., 2002). Variabilities in the strength, position, wave amplitudes, and location of the CPV are well-known to be linked directly to variations in surface environmental features such as surface air temperature and wind (van den Broeke and van Lipzig, 2002; Moron et al., 2018), precipitation (Srinivas et al., 2018), sea surface temperature (Frauenfeld et al., 2005), ocean salinity (Chen et al., 2018), water vapor transport (Wang and Ding, 2009), air mass properties (Vanos and Cakmak, 2014), storm tracks (Kidston et al., 2015), ozone (Glovin et al., 2016), sea ice extent (Orme et al., 2017), aerosol distributions (Bartlett et al., 2018), and atmospheric blocking (Altenhoff et al., 2008; Tyrlis and Hoskins, 2008), just as Earth surface topography is associated directly to the steering circulation (Tang and Chan, 2016).

In an early study, Angell and Korshover (1977) noted that the 300-hPa annual-averaged NHCPV area increased from 1970 to 1975 despite simultaneously shrinking over time in winter. Angell (1998, 2006) later suggested that the 300-hPa-NHCPV area was decreasing over the 1963–2001 period, especially in the Western Hemisphere, at a rate of 1.5% per decade. Angell (1998) demonstrated that the most intense 300-hPa-NHCPV contraction began around 1990. By contrast, Davis and Benkovic (1992, 1994) noted an expansion of the 500-hPa January NHCPV from 1966 to 1990, primarily resulting from intensified regional troughing over the North Pacific Ocean and eastern North America and concurrent warm air mass intrusion into Alaska and western Canada. Burnett (1993) found similar results for the January NHCPV after the mid-1960s, with areal growth due to expansion along the Pacific and eastern North America/Atlantic sectors, but with contraction commencing in the late 1980s. Frauenfeld and Davis (2003) investigated temporal trends of NHCPV area at multiple constant-pressure levels over the 1949–2000 period; at all levels they found significant NHCPV expansion until 1970 and areal contraction afterward, with the primary areas of expansion/contraction over Asia, Europe, and North America, and less variability over the Northern Hemisphere oceans. Trends were stronger in the upper troposphere than near the surface (Frauenfeld and Davis, 2003), likely because of the complexity of the surface influence. Using threshold temperatures at the 850 hPa level as the indicator, Martin (2015) observed a shrinking of the Northern Hemisphere cold pool over time.

While all of the above research delineated the NHCPV area using a pre-determined isohypse, as recommended by Frauenfeld and Davis (2003), Thompson and Solomon (2002) applied vertical gradients of geopotential heights at the relatively coarse network of radiosonde-based stations to define the NHCPV. Bushra and Rohli (2019) delineated the CPV's leading edge at the sharpest latitudinal gradient of 500-hPa geopotential height.

Although the NHCPV's area has been studied for several decades, analysis of CPV shape had been largely restricted to reporting the relative area within various quadrants about the pole until Rohli et al. (2005), Wrona and Rohli (2007), and Bushra and Rohli (2019) borrowed from watershed hydrologists' drainage basin metrics (Chorley et al., 1984) by using a “circularity ratio” (*R*_*c*_), ranging from 0 (i.e., infinitely amplified ridges and troughs) to 1.0 (i.e., perfectly circular, west-to-east-flowing CPV). CPV sinuosity (i.e., ratio of CPV length to the length of the corresponding parallel of latitude segment) has also been analyzed for the 500-hPa level in the North American sector (Vavrus et al., 2017) and at the hemispheric scale (Cattiaux et al., 2016; Di Capua and Coumou, 2016), using a fast Fourier transform (FFT) approach (Screen and Simmonds, 2013), and for potential-vorticity-based identification of Rossby wave initiation (Röthlisberger et al., 2016). However, despite these and a few other important studies (e.g., Zakinyan et al., 2016), there remains a need for examining and interpreting the CPV shape.

Analyzing temporal trends in the NHCPV and its *R*_*c*_ may reveal important features of hemispheric-scale response to surface warming. While air temperatures are increasing globally, the warming trend of the past several decades may be most pronounced in the high latitudes, especially in the Arctic (Holland and Bitz, 2003; Pithan and Mauritsen, 2014; Post et al., 2019). This “Arctic amplification” decreases the lateral temperature gradient and affects the tropics-to-poles transfer of energy in both the atmosphere (e.g., Cohen et al., 2014) and ocean (e.g., Rahmstorf et al., 2015; Praetorius, 2018). Arctic amplification has been proposed to weaken the zonal winds, which is connected to enhanced Rossby wave ridging and troughing and support for severe weather by increasing areas of ULD and CVA. Model-based research has posited that these meridional meanders will increase into the future (Francis and Vavrus, 2012, 2015; Peings and Magnusdottir, 2014), but Cattiaux et al. (2016) disagreed, with Vavrus (2018) summarizing the conflicting evidence and lamenting the lack of consensus.

Cyclical variation in the broad-scale steering circulation has included analysis of the energy distribution of the atmospheric motion and waves. This oscillation can be explained through their linearized/non-linearized energy transfer spectra with Fourier analysis over space and time. Understanding of the relationship between wave number and frequency, and variability of magnitude and phase could aid understanding of the dynamics of the energetics influencing surface weather. Hayashi (1974) performed space-time cross spectrum analysis to understand the effect of tropical disturbances on the general circulation. Chen et al. (1981) simulated winter wind fields over a 90-day period by applying modified Fourier analysis to characterize the wavenumber-frequency spectra. Hayashi (1982) used space-time spectral analysis to understand broad-scale atmospheric waves. Higuchi et al. (1999) employed multiresolution Fourier transform spectral analysis to resolve the temporal structure of the variation of the North Atlantic Oscillation (NAO; van Loon and Rogers, 1978; Wallace and Gutzler, 1981; Barnston and Livezey, 1987; Hurrell, 1995), a component of the NHCPV, in terms of various frequency components. Torrence and Compo (1998) explained the implications of wavelet analysis in the atmospheric sciences using the example of the El Niño–Southern Oscillation (ENSO; Philander, 1990) time series. Pelletier (1998) explored local variability of the high-frequency component of the temperature time series using a power spectrum. Ghil et al. (2002) applied time series analysis by treating the atmosphere as a non-linear dynamic system. Rodionov (2004) applied sequential algorithm in time series data to detect regime shifts and track magnitude changes. More recent research has included the examination of the daily 500-hPa geopotential height data of the Southern Hemisphere to analyze the space-time frequency characteristics of the expansion coefficients (Sun and Li, 2012).

A comprehensive analysis of the spatiotemporal cyclical nature of the NHCPV area and *R*_*c*_ remains lacking. Implications of the linear regression model represent the temporal trend over the time domain while the FFT extracts information on the variability of the magnitude and oscillations by analyzing the frequency variability.

## Purpose

The purpose of this research is to evaluate temporal variations of the NHCPV's area and *R*_*c*_ at the 500-hPa geopotential height level. Time-series analysis will identify the periodicity/frequency characteristics and variability over time. Analysis of the intra- and inter-annual variabilities of the NHCPV area and *R*_*c*_ provides an index of the relationship between the atmospheric steering flow and surface warming, and it also contextualizes recent results (Bushra and Rohli, 2019). Analyzing the intra- and inter-annual variability of the NHCPV shape is important not only as a potential characterization of the response to Arctic amplification, but also as a possible indicator of baroclinic instability associated with severe extratropical weather in future work. Analysis of NHCPV area and *R*_*c*_ also informs studies on extratropical severe weather that rely on the juxtaposition of tropical and polar air masses (e.g., Overland et al., 2015).

## Data and methods

To facilitate comparison of results to Bushra and Rohli (2019, 2021a,b), daily 500-hPa geopotential height data are obtained from the National Centers for Environmental Information (NCEI)/National Center for Atmospheric Research (NCAR) Reanalysis 2 project (Kanamitsu et al., 2002). The spatial resolution of this data set allows for delineation of longwave flow while filtering out shortwave flow, which assists in overcoming the computational challenges from daily CPV shapes perforated by cutoff lows, omega blocks, and Rex blocks (Lupo, 2021). At each 2.5° increment of longitude globally from 20°N to the North Pole, the daily 500-hPa NHCPV is delineated over the 1979–2017 period using the method described more fully in Bushra and Rohli (2019). Specifically, the NHCPV polygon is formed by connecting the dots representing the latitude of the steepest gradient of 500-hPa geopotential height for each meridian of longitude around the Northern Hemisphere. This approach circumvents the tenuous assumption that the leading edge of the CPV follows the same isohypse across space, despite differences in topographic, marine, and air mass influence. After Bézier smoothing (Jekeli, 2005), the NHCPV area is then measured, and *R*_*c*_ is calculated as


$$R_{c}\;=\;\frac{A}{A_{c}}$$

where *A* represents the area enclosed within the NHCPV and *A*_*c*_ denotes the area of the circle with the same perimeter as the NHCPV on that day. Because the calculation of segment length makes *R*_*c*_ resolution-dependent, it is important that the same data used in earlier calculations of NHCPV properties (Bushra and Rohli, 2019, 2021a,b) are used here.

Statistical regression is then used to analyze the linear trend of the NHCPV's area and *R*_*c*_ for the 500-hPa level for the entire time period and subperiods identified *via* breakpoint analysis (Bai and Perron, 2003; Muggeo, 2008). The number of breakpoints identified depends on the testing and deviations from stability in the linear regression models, where the coefficients shift from one stable regression relationship to a different one (Bai, 1994, 1997a,b; Bai and Perron, 1998). If there are m breakpoints, the number of segments in which the regression coefficients are constant will be m+1.

To remove high-frequency noise, a Butterworth (Butterworth, 1930) low-pass filter is applied at a cutoff point of 3.65 year^−1^ to the dataset, before applying FFT (Welch, 1967). Then, Fisher's Exact G Test for Multiple (Genetic) Time Series, or “fisher.g.test” (Ahdesmaki et al., 2021), is applied to the NHCPV's area and *R*_*c*_ data for the entire record and segmented time periods resulting from the breakpoint analysis to confirm that remaining noise is not problematic. Any *p*-values of < 0.001 support rejection of the null hypothesis of significant presence of white noise.

The presence of lag-1 autocorrelation between two consecutive observations is indicative of red noise (Schulz and Mudelsee, 2002) and has a power spectrum weighted toward low frequencies (Ghil et al., 2002). Application of the Durbin-Watson (D-W) test (Savin and White, 1977) with the test statistic of <2 (*p*-value < 0.001) for all data series confirms the presence of lag-1 autocorrelation in the data sets. To confirm that the resultant power spectra are not from red noise, the Cochrane–Orcutt (C-O) method (Montgomery et al., 2012) is used to remove any such autocorrelation to make the data sets independent in time (Vanhatalo and Kulahci, 2016). Again, application of D-W test with a test statistic of >2 (*p*-value < 0.001) suggests that lag-1 autocorrelation (i.e., red noise) is removed successfully from each data sets.

Finally, FFT is applied for spectral analysis. Spectral analysis identifies the space-time Fourier component of the NHCPV properties to evaluate the periodic characteristics of the area and *R*_*c*_ time series data for all time periods by decomposing their time series signal into the frequency domain (i.e., quantifying their magnitude of variations at different frequencies).

## Results and discussion

### Long-term trends

**Figure 1:**
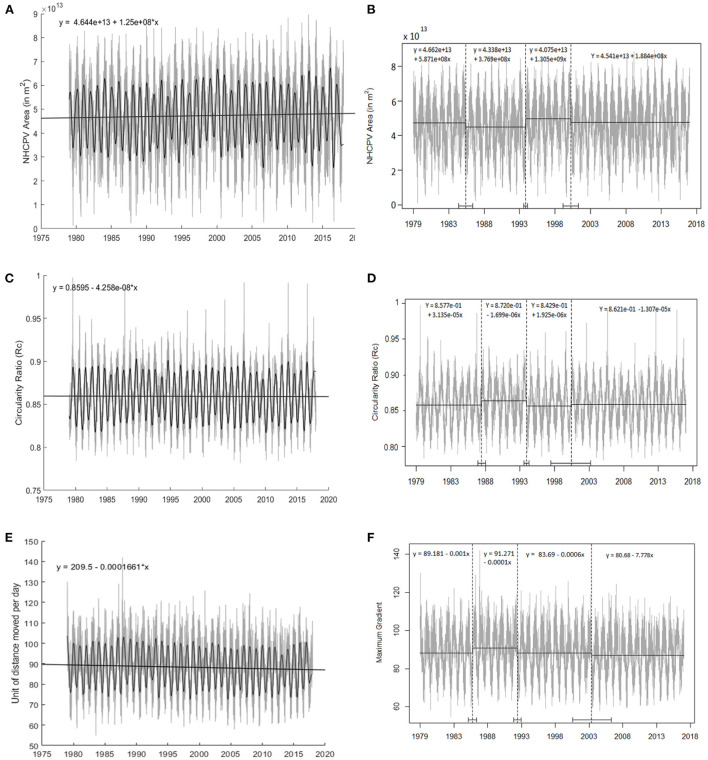
**(A)** Statistically significant (*p* < 0.001) linearly increasing trend in daily 500-hPa NHCPV area over the 1979–2017 period. The smoothed black line, from Butterworth low-pass filtering, shows the annual cycle. **(B)** Linear regressions from breakpoint analysis of four subperiods of 1979–1986, 1986–1994, 1994–2001, and 2001–2017 for NHCPV area (*p*-values shown in Table 1) with the 95% confidence interval for the breakpoint positions shown along the x-axis and in Table 1. **(C)** As in **(A)** but showing statistically insignificant (*p* = 0.445) linearly decreasing trend in 500-hPa *R*_*c*_. **(D)** As in **(B)**, but for four subperiods of 1979–1988, 1988–1994, 1994–2001, and 2001–2017 for NHCPV *R*_*c*_. **(E)** As in **(A)**, but showing statistically significant (*p* = 0.001) linearly decreasing trend in 500-hPa mean maximum daily north-south 500-hPa geopotential height gradient. **(F)** As in **(B)**, but for four subperiods of 1979–1986 (*p* = 0.016), 1986–1993 (*p* = 0.718), 1993–2004 (*p* < 0.001), and 2004–2017 (*p* < 0.001) for NHCPV gradients.

Linear regression analysis reveals that over the 1979–2017 time period, the area enclosed within the 500-hPa NHCPV shows a significant temporal increase (125 km^2^ day^−1^; *p* < 0.001; Figure 1A) despite coincident increases in global mean surface temperature (Folland et al., 2018). A warming world might have been expected to be characterized by a shrinking cold pool as represented by the NHCPV. Time series breakpoints are identified at a 95% confidence level in 1986, 1994, and 2001, with NHCPV area increasing significantly within all four segmented periods (Figure 1B), but with the 1979–1986 segment showing an increasing area only at a 90% confidence level (Table 1). The 95% confidence interval of each breakpoint location is shown in Figure 1B and Table 1. The greatest rate of NHCPV areal increase (1,305 km^2^ day^−1^) occurred over the 1994–2001 period, perhaps because of cooling at the end of the extended El Niño event in the early 1990s, which enlarges the Pacific sector of the NHCPV while shrinking the American sector (Frauenfeld and Davis, 2000). The eruption of Mount Pinatubo in the Philippines apparently was not an important factor, as it has been shown to be associated with shrinking, while strengthening, the NHCPV (Otterå, 2008). The lowest rate of 188 km^2^ day^−1^ occurred over the 2001–2017 period, possibly corresponding to shift in the Pacific Decadal Oscillation (PDO; Newman et al., 2003) to a cold phase (National Oceanic Atmospheric Administration, 2021), which might partially offset the tendency for NHCPV expansion, particularly in the Pacific basin. Both of these hypotheses invite further investigation, but the time series is not sufficiently long to identify multiple cycles of influence from either the Atlantic Multidecadal Oscillation (Kerr, 2000) or the PDO (Rohli et al., 2022). Note that because of the abrupt downward shift in area at the breakpoints, the minimum rate of areal growth in subperiods still exceeds the mean temporal increase over the entire time series.

**Table 1:** Regression line with significance of slope, and 95% confidence interval of breakpoints, for NHCPV area and *R*_*c*_.

**Area**			***R***_*****c*****_		
**Sub-periods**	**Regression line (** ***p*** **-value)**	**Breakpoints (confidence interval)**	**Sub-periods**	**Regression line (** ***p*** **-value)**	**Breakpoints (confidence interval)**
01/01/1979–5/26/1986	y = 4.662e+13 + 5.871e+08x (*p* = 0.086)		01/01/1979–6/4/1988	y = 8.577e-01 + 3.135e-05x (*p* = 0.061)	
5/27/1986–10/25/1994	y = 4.338e+13 + 3.769e+08x (*p* = 0.0413)	5/27/1986 (6/2/1985 −5/19/1987)	6/5/1988– 12/1/1994	y = 8.720e-01 – 1.699e-06x (*p* = 0.031)	6/5/1988 (11/22/1987–1/13/1989)
10/26/1994–3/24/2001	y = 4.075e+13 + 1.305e+09x (*p* = 0.002)	10/26/1994 (7/30/1994 −1/25/1995)	12/2/1994–5/28/2001	y = 8.429e-01 + 1.925e-06x (*p* = 0.017)	12/2/1994 (3/26/1994–7/17/1995)
3/25/2001–12/31/2017	y = 4.541e+13 + 1.884e+08x (*p* = 0.040)	3/25/2001 (2/2/2000 −4/13/2002)	5/28/2001– 12/31/2017	y = 8.621e-01 – 1.307e-05x (*p* = 0.068)	5/28/20013/12/1997–2/3/2003)

Another possible explanation for the temporal increase in NHCPV area may be that in the warming world during these decades, the leading edge of the NHCPV may be becoming less distinct, leading to cases in which the subtropical jet (STJ) could possibly mistakenly be delineating the CPV, leading to an increasingly larger CPV area. This is a particular concern in the summer months, because the consideration of data points only poleward of 20°N would limit the possibilities for inclusion of the STJ in the cold season. The large areal extent of a STJ-based NHCPV of the warm season may overcompensate for the shrinking polar-front-based, cold-season NHCPV, leading to net NHCPV expansion.

Linear regression analysis of the 500-hPa NHCPV's *R*_*c*_ reveals no significant trend (−4.258 × 10^−8^ day^−1^; *p* = 0.445) over the entire time series (Figure 1C). Four time series segments are also identified for the 500-hPa NHCPV *R*_*c*_, with breakpoints in 1988, 1994, and 2001 and the 95% confidence interval of each breakpoint location shown in Figure 1D and Table 1. These breakpoints are similar to those for the area analysis, probably suggesting that the trend of the undulation of the ridges and troughs is related to the NHCPV area, and that regime shifts for NHCPV area and circularity happen simultaneously.

In the 1979–1988 and 1994–2001 segments, *R*_*c*_ increases significantly (at 90 and 95% confidence levels, respectively) with the highest increasing rate of 0.02 × 10^−8^ day^−1^ over the latter period. However, these subperiods are interrupted by intervals of decreasing trends for 1988–1994 and 2001–2017 (at 95 and 90% confidence levels, respectively) with the greatest decreasing rate of −0.017 × 10^−8^ day^−1^ over the earlier period.

The apparently fluctuating but overall temporally increasing area of the CPV amid temporally warming conditions could leave less volume for the remaining cold air pool, leading to increasing circularity. It could also be related to the fact that, in contrast to the cold pool analysis of Martin (2015) which only considers DJF, the analysis here considers the entire year. But the trend identified here could be counteracted by a tendency for increased wave amplitudes amid a decreasing equator-to-pole temperature gradient, as observed by Martin (2021), as the forces for geostrophic flow weaken, in a manner analogous to the increased meandering of a stream amid a gentler gradient near its mouth. The net result would be a relationship between area and circularity but with no overall linear temporal trend in *R*_*c*_ despite a temporally decreasing NHCPV area.

This result suggests that the 500-hPa NHCPV's shape may be characterized by alternating increases and decreases in Rossby wave amplification over time. Decreasing circularity may be expected to occur in an environment of weakening equator-to-pole gradients of 500-hPa geopotential height and weakening zonality of winds, which may in turn amplify the Rossby waves (Francis and Vavrus, 2012), providing for increasing frequency and magnitude of weather extremes. But this pattern is “reset” eventually, as a trend for increasing circularity sets in for a period of time before the former pattern is re-established. The areal expansion contrasts with the notion that the 500-hPa NHCPV area would decrease over time as the cold pool retreats poleward in response to surface warming and instead suggests that other factors may be driving the result.

Therefore, in a follow-up analysis, it is confirmed that the daily-averaged 500-hPa geopotential height gradient that demarcates the leading edge of the NHCPV is indeed decreasing significantly, at a rate of 0.1661 m km^−1^ day^−1^ (*p* < 0.001) over the study period (Figure 1E). This value is calculated from the trendline (y = 89.44–1.661 × 10^−4^ (note here that the units of the slope of the equation are m m^−1^ day^−1^); *p* < 0.001) of the 14,245 daily mean values of the 144 daily longitudinal maximum 500-hPa daily geopotential height difference (in m) at a given longitude occurring across the horizontal distance between two adjacent grid points 2.5° of latitude (or 279.23 km) apart. This weakening gradient may be a stronger symptom of Arctic amplification than the NHCPV area. Decreasing trends within each of the four subperiods of the study period are also apparent but with different levels of statistical significance (Figure 1F).

### Seasonal cycle

**Figure 2:**
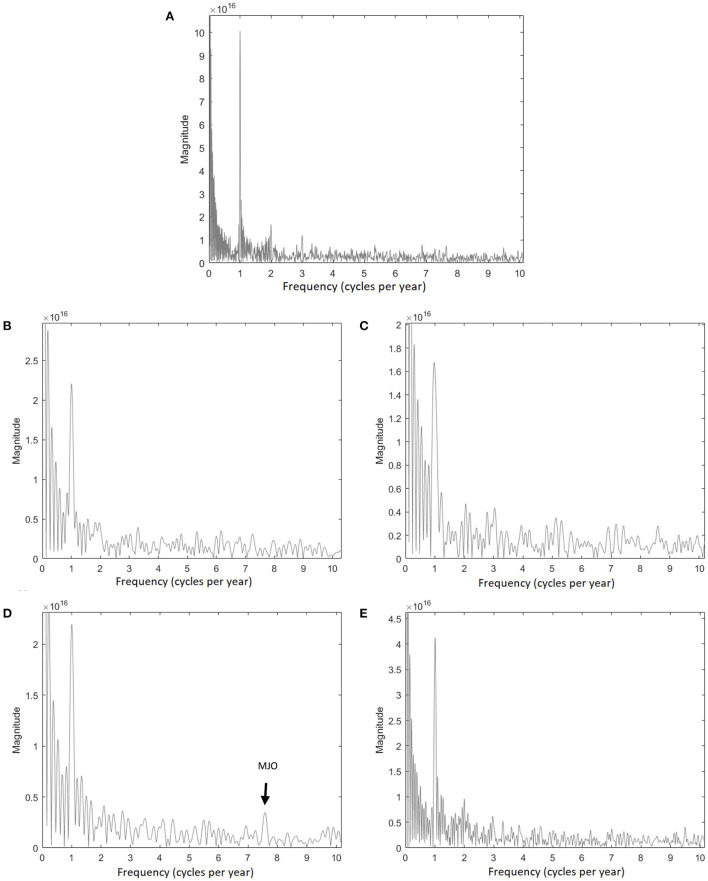
Magnitudes of the power spectra of the daily NHCPV area over **(A)** 1979–2017, **(B)** 1979–1986, **(C)** 1986–1994, **(D)** 1994–2001, and **(E)** 2001–2017 periods. The peaks indicate the magnitude of the high-frequency variability. The highest peaks at 1 cycle year^−1^ indicate an annual cycle for all periods.

The power spectrum allows an assessment of the frequency and amplitudes of the 500-hPa NHCPV characteristics. The NHCPV area has a distinct annual cycle suggestive of the variation of solar radiation over each year in the Northern Hemisphere due to the tilt of Earth's axis of revolution and a semiannual cycle perhaps reflective of the cold-warm seasonal flow, over the complete study period (1979–2017). However, the magnitude of the annual cycle varies over the subperiods with an indistinct or weaker semiannual cycle (Figures 2A–E). The semiannual signal among these daily standardized values suggests that z-scores of the area might offer at least some degree of understanding for the z-scores of area 6 months into the future. FFT also reveals a high frequency intra-annual cycle of ~7.5 year^−1^ but only over the 1994–2001 period (Figure 2D). This frequency matches that of the 40–60-day oscillation, also known as the Madden-Julian Oscillation (MJO; Madden and Julian, 1994; Henderson et al., 2016; Gollan and Greatbatch, 2017), which is a tropical eastward-propagating sequence of convective activity that was identified *via* zonal wind anomalies in the tropical Pacific Ocean (Madden and Julian, 1971). Given the demonstrated link between tropical and extratropical circulation anomalies (e.g., Lau et al., 2012), a link between the MJO and NHCPV is a topic that warrants future research. The 1994–2001 subperiod showed the strongest increasing trend in NHCPV area.

**Figure 3:**
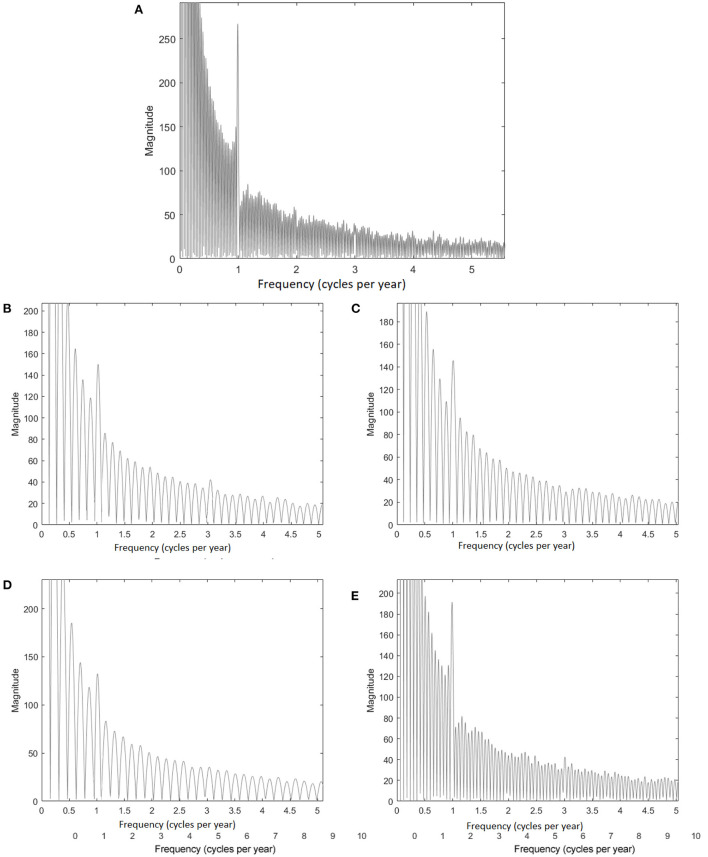
As in Figure 2, but for *R*_*c*_ over **(A)** 1979–2017, **(B)** 1979–1988, **(C)** 1988–1994, **(D)** 1994–2001, and **(E)** 2001–2017 periods.

FFT analysis for the NHCPV *R*_*c*_ also shows a distinct annual cycle over the 1979–2017 period, but with less evidence of a semiannual cycle (Figures 3A–E). The annual signal has similar strength over the 1979–1988, 1988–1994, and 1994–2001 subperiods (Figures 3B–D), while the magnitude of 2001–2017 (Figure 3E) is greater. The smaller amplitudes in the earlier stage of the time series for the three subperiods imply that the shape of the CPV had less association to the annual cycle of solar radiation than in the 2001–2017 subperiod (i.e., the warmest decades). In addition, the 1979–1988 and 2001–2017 periods (Figures 3B, E) show a secondary cycle at 3 year^−1^.

The reason for this cycle for only these two subperiods is unclear and deserves future research consideration. However, the NAO and the related Arctic Oscillation (Thompson and Wallace, 1998, 2000) are likely responses to the forcing mechanisms as they have been shown to be closely linked to the NHCPV, even at the daily time scale (Bushra and Rohli, 2021a).

## Summary and conclusions

Analysis of daily variability of area and circularity of the NHCPV defined by the sharpest 500-hPa geopotential height gradient offers potential for improved understanding of the mid-latitude circulation. In this research, long-term changes in the area and *R*_*c*_ of the 500-hPa NHCPV are presented for the 1979–2017 period of record.

Linear regression analysis suggests that the NHCPV area has increased significantly over time, including temporal increases over each subperiod of the time series as revealed by breakpoint analysis. Concurrent with the areal change is a temporally significant trend toward a reduction in the 500-hPa geopotential height gradient that separates the polar cold pool from the temperate atmosphere and delineates the leading edge of the NHCPV. Thus, the distinctiveness of the NHCPV blurs over time, which may impact the trends and the NHCPV's covariation with surface weather. No linear change in *R*_*c*_ was observed over 1979–2017 periods, but the four subperiods are similar to those for the area analysis, suggesting that the regimes that govern area also govern circularity. Adjacent subperiods of *R*_*c*_ show reversing trends and different significance levels (i.e., 90–95%).

Application of the power spectrum to a low pass filtered time series reveals that both NHCPV area and *R*_*c*_ have distinct annual cycles but with varying amplitude. FFT also identifies intra-annual cycles for subperiods.

Collectively, these results are important indicators of the association of global temperature change at varying time scales with the upper-level steering circulation. Analyzing CPV characteristics reveals important information about how hemispheric circulation may be related to the surface environment and may have associations to extreme atmospheric events. Analysis of the intra-annual variability of the NHCPV area provides a tangible index of the link between atmospheric steering flow and the observed surface warming.

## Data availability statement

The raw data supporting the conclusions of this article will be made available by the authors, without undue reservation.

## Author contributions

NB developed the detailed methodology, collected and analyzed the data, and developed the initial text. RR helped develop the methodology and edited early and late drafts of the text. CL and PM conceptualized the methodologies and revised the text, provided feedback, and revised the text. RM helped to organize the paper, prepared the figures, and addressed reviewers comments. All authors contributed to the article and approved the submitted version.

## Supplementary material

Methods associated with the generation of the data used in this research to characterize the NHCPV properties are described in more detail in Bushra and Rohli (2019). The gridded data set was obtained from the National Centers for Environmental Prediction (NCEP)/Department of Energy (DOE) Reanalysis 2 project. The data are available at https://www.esrl.noaa.gov/psd/data/gridded/data.ncep.reanalysis2.pressure.html.
